# Department of Dermatology

**Published:** 1893-06

**Authors:** Isadore Dyer

**Affiliations:** Tulane University, N. O.


### DEPARTMENT OF DERMATOLOGY.

[Notes by Dr. Isadore Dyer, Tulane University, N. O.]

The lather of a Calomel soap, applied and left on the skin, is a recently advanced treatment of syphilis.

---

For hyperidrosis with fetor, Dr. Long suggest ℞ Ext. white agaric, gr. viii to xv, alcohol, ℥ii. Sig. To be painted on.

---

An Austrian physician recommends dermatol after cauterization with nitrate of silver, to relieve the pain. It is to be dusted on.

---

Dr. J. Hutchinson reports a case of leprosy cured under long arsenic treatment and abstinence from fish diet. The patient had enjoyed three years immunity since the last lesions had disappeared.

---

Pilocarpine hydrochlorate is suggested as efficacious in the treatment of elephantiasis abrabrum. From one-sixth to one-third of a grain is daily injected into the tissues involved.—*Dr. Poulet*, *Semaine Medicale*.

---

Dr. Leloir advocates warm baths and prolonged immersion as a therapeutic measure in acute lichen planus. The itching is promptly relieved and the inflammation reduced.—*Transactions French Dermatological Society.*

---

Europhene has been used by Dr. J. Goldschmidt (Madeira) in the treatment of Leprosy. A five per cent. solution in olive oil was rubbed in three times a day for five minutes at a time. Injections of the oil were also made into the nodules.

---

PEROXIDE OF HYDROGEN AS A PRESERVATIVE AGAINST SYPHILIS AND SOFT CHANCRES.—Dr. Z. Krowczynski, senior surgeon to the special department for syphilitic and skin diseases of the Lemberg hospital (Galicia), has performed a series of very interesting experiments from which it would appear that the use of a slightly acid solution of peroxide of hydrogen may prove to be an excellent preservative against syphilis and soft chancres.

The experiments consisted in the inoculation of patients suffering from syphilis or chanchroidal ulcerations, in some cases with the unaltered pus from soft chancres, and in others with the pus after admixture in a watch glass with the following solution:

℞ Hydrochloric acid. . . . . . . . . . . . gtt. x
3 % solution of peroxide of hydrogen . . ℥iv.

Dr. Krowczynski selected the peroxide of hydrogen for his experiments because of its powerful antiseptic properties and of its freedom from all caustic action. It was thought desirable to add a small quantity of hydrochloric acid to increase the destructive power of the fluid as regards the virus of the above mentioned affections, previous researches having satisfied the investigator that the secretions from chancroidal and syphilitic sores are always alkaline in reaction. The addition of the acidified peroxide of hydrogen solution to the secretion from soft chancres always gives rise to a brisk effervescence.

The patients were inoculated on the forearm, after carefully scarifying a limited area of skin with the point of a knife. The site of inoculation was first washed with soap and water, and wiped dry after being rendered aseptic with a 3% solution of carbolic acid.

The results of the experiments have so far proved very satisfactory. Fifteen inoculations were made with the pure pus, all which gave positive results, that is to say on the third day after inoculation, a small pustule appeared, which soon became surrounded by a zone of inflammation. The pustule ran the usual course of typical soft chancres. On the contrary, out of fifteen inoculations with the pus after admixture with the peroxide of hydrogen solution, in fourteen the results were absolutely negative. In the fifteenth case, a patient suffering from late syphilitic manifestations, an ulcer formed at the site of inoculation on the sixth day, but it presented none of the ordinary appearances of soft chancres.

Lastly, Dr. Krowczynski has had the opportunity of inoculating two medical men who had never had syphilis, and who volunteered to submit to the experiment, with syphilitic virus previously mixed with the acidified peroxide of hydrogen solution. The inoculations were made through the scarified skin of the forearm as before, in the one case with the secretion from a specific chancre, and in the other with that of a mucous tubercle. The parts were not covered until they were quite dry, after which they were kept in cotton-wool for three days. In both cases the results were negative.

Although the facts observed by Dr. Krowczynski, as he himself admits, are far from being conclusive, owing to the small number of cases experimented upon, especially in respect of syphilis, we think that these experiments present sufficient interest to justify us in describing that at some length. The question is doubtless one of the greatest practical importance, for a very great advance would be made in the prophylaxis of venereal diseases if the risk of infection with syphilis or soft chancres could be done away with by the external application of peroxide of hydrogen or some other fluid after a doubtful connection.

It is obvious that the antiseptic power of the peroxide of hydrogen solution depends on its strength. Dr. Krowczynski employed a comparatively weak solution (3%) because he was unable at the time to procure a more concentrated preparation.

